# Efficacy of eribulin for metastatic breast cancer based on localization of specific secondary metastases: a post hoc analysis

**DOI:** 10.1038/s41598-020-66980-0

**Published:** 2020-07-08

**Authors:** Joyce O’Shaughnessy, Javier Cortes, Chris Twelves, Lori J. Goldstein, Karenza Alexis, Ran Xie, Carlos Barrios, Takayuki Ueno

**Affiliations:** 1 Baylor University Medical Center, Texas Oncology and US Oncology, Dallas, TX USA; 2 IOB Institute of Oncology, Quironsalud Group, Madrid and Barcelona & Vall d’Hebron Institute of Oncology (VHIO), Barcelona, Spain; 3 Leeds Institute of Medical Research at St James’s and Leeds Teaching Hospitals Trust, Leeds, UK; 4 Fox Chase Cancer Center, Philadelphia, PA USA; 5 Eisai Inc., Woodcliff Lake, NJ USA; 6 Latin American Cooperative Oncology Group (LACOG), Porto Alegre, Brazil; 7 Breast Oncology Center, The Cancer Institute Hospital of the Japanese Foundation for Cancer Research, Tokyo, Japan

**Keywords:** Breast cancer, Predictive markers, Prognosis, Risk factors

## Abstract

Prior pooled analysis of eribulin studies (301 and 305) indicated eribulin prolonged overall survival (OS) in patients with locally advanced/metastatic breast cancer (MBC) regardless of visceral or nonvisceral disease. This hypothesis-generating post hoc analysis examined the efficacy of eribulin according to the location of metastatic sites at baseline in 1864 pretreated patients with locally advanced/MBC from studies 301 and 305. Analyses included OS, progression-free survival (PFS), and objective response rate; OS and PFS were also analyzed according to estrogen-receptor status. Eribulin appeared efficacious in patients with locally advanced/MBC, irrespective of the location of metastases at baseline. A nominally significant difference in OS in favor of patients randomized to eribulin compared with control in patients with bone, lymph node, and chest wall/breast/skin metastases at baseline was observed. Additionally, a difference in OS was also seen in patients with liver metastases randomized to eribulin versus control (median: 13.4 versus 11.3 months, respectively; hazard ratio, 0.84 [95% CI: 0.72, 0.97]). Results of this exploratory analysis suggest that eribulin may be efficacious for the treatment of locally advanced/MBC for patients with bone, liver, lung, lymph node, and chest wall/breast/skin metastases.

## Introduction

Metastatic breast cancer (MBC) is a heterogenous disease, with metastases commonly located in soft tissue (ie, skin, lymph nodes, and the contralateral breast), bone, and visceral organs (ie, lungs, pleura, peritoneum, liver, and brain)^1^. Although treatment for MBC has steadily improved, few agents have been shown to prolong survival and MBC remains essentially incurable with a median overall survival (OS) of 2–3 years^2,3^. Moreover, patients with visceral metastases typically have a worse prognosis than patients with nonvisceral metastases^2,4–6^.

Eribulin is a synthetic analogue of halichondrin B, originally isolated from the marine sponge *Halichondria okadai*^7^. The antitumor activity of eribulin is driven by a distinct mode of interaction with microtubules, in which eribulin inhibits the growth phase but not the shortening phase^8–11^. *In vitro* and preclinical studies suggest that eribulin may also have nonmitotic mechanisms including vascular remodeling, reversal of the epithelial-to-mesenchymal (EMT) transition, and inhibition of cancer cell migration/invasion^12–14^; some of these noncytotoxic effects have also been demonstrated clinically^15^. In the United States, eribulin is indicated for the treatment of patients with MBC who have previously received ≥2 chemotherapeutic regimens for the treatment of metastatic disease—prior therapy should have included an anthracycline and a taxane in either the adjuvant or metastatic setting. Additionally, approval for eribulin in MBC has been extended to the second-line metastatic setting in other geographic areas, including the European Union.

The efficacy of eribulin has been demonstrated in several phase 3 studies (Studies 305, 301, and 304) in patients with locally advanced or MBC that had been more heavily pretreated (Studies 305 and 304)^16,17^ or less heavily pretreated (Study 301)^18^. In the studies included in our analysis (Studies 305 and 301), pretreatment was required to include a taxane and an anthracycline^16,18^. In Study 305 (EMBRACE; NCT00388726), the primary end point was achieved with eribulin significantly improving OS compared with treatment of physicians’ choice (TPC; hazard ratio [HR], 0.81 [95% CI: 0.66, 0.99]; *P* = 0.041)^16^; an updated OS analysis confirmed this benefit in OS for eribulin (13.2 months versus 10.5 months; HR, 0.81 [95% CI: 0.67, 0.96]; *P* = 0.014)^16^. Study 301 compared eribulin with capecitabine (NCT00337103); eribulin was associated with a nonsignificant increase in OS compared to capecitabine (15.9 months versus 14.5 months, respectively; HR, 0.88 [95% CI: 0.77, 1.00]; *P* = 0.056)^18^. In both studies, the toxicity of eribulin was manageable and comparable to that of other chemotherapeutic agents used in this setting^16,18^.

Although eribulin is widely used in patients with previously treated MBC, its efficacy according to metastatic organ sites has not been well characterized. However, in one earlier subgroup analysis of Studies 305 and 301 combined^19^, eribulin improved OS compared with TPC/capecitabine in patients with visceral (median OS, 14.3 versus 12.2 months; HR 0.89; *P* = 0.037) and nonvisceral (median OS, 18.8 versus 16.6 months; HR 0.72; *P* = 0.045) disease; moreover, greater benefit was seen with eribulin compared with TPC/capecitabine in patients with >2 involved metastatic organ sites (median OS, 13.1 versus 10.5 months; HR 0.77; *P* < 0.001)^19^. In this current post hoc pooled exploratory analysis of Studies 305^16^ and 301^18^ we assess OS, progression-free survival (PFS), and objective response rate (ORR) according to organ sites of metastases at baseline for patients randomized to receive eribulin versus TPC/capecitabine. Additionally, we present the percentage change in the sum of the diameters of target lesions from baseline to postbaseline nadir according to sites of metastases (ie, waterfall plots) and OS and PFS according to metastatic site and estrogen-receptor (ER) status.

## Methods

### Patients

Patient eligibility criteria for Study 305^16^ and Study 301^18^ have been published. Briefly, eligible patients were women ≥18 years of age with histologically or cytologically confirmed breast cancer, an Eastern Cooperative Oncology Group performance status of 0–2, and adequate bone marrow, liver, and renal function. In Study 305, patients were previously treated with 2–5 chemotherapy regimens (of which ≥2 were for locally recurrent breast cancer or MBC)^16^, and in Study 301, patients were previously treated with ≤3 chemotherapy regimens (of which ≤2 were for advanced and/or metastatic disease)^18^.

The source Studies 305 and 301 were conducted in accordance with the World Medical Association Declaration of Helsinki, ethical standards of the responsible committee on human experimentation (institutional and national), and guidelines of the International Conference for Harmonization/Good Clinical Practice. Informed consent was obtained from all individual participants included in the studies and both studies were approved by ethics committees (Study 305: The New York Presbyterian Hospital-Weil Medical College of Cornell University Committee on Human Rights in Research [for the lead investigator in the United States]; Study 301: Committee for the Protection of Human Subjects [for the lead investigator in the United States]).

### Study design and treatment

Studies 305 and 301 were phase 3, multicenter, open-label, randomized trials that compared the efficacy of eribulin with TPC (Study 305) or capecitabine (Study 301) in women with locally advanced/MBC^16,18^. In Study 305, patients were randomly assigned (2:1) to either eribulin mesylate (1.4 mg/m^2^ [equivalent to eribulin 1.23 mg/m^2^ when expressed as a free base]; administered intravenously over 2–5 minutes on days 1 and 8 of a 21-day cycle) or TPC (any single-agent chemotherapy, or hormonal or biological therapy approved for the treatment of cancer and administered according to local practice; radiotherapy; or symptomatic treatment alone). In Study 301, patients were randomly assigned (1:1) to either eribulin mesylate (1.4 mg/m^2^; intravenously over 2–5 minutes on days 1 and 8 of a 21-day cycle) or capecitabine (1.25 g/m^2^; orally twice daily on days 1–14 of a 21-day cycle). Treatment in both studies continued until disease progression, unacceptable toxicity, or patient/investigator request to discontinue.

### Study end points

The primary end point of Study 305 was OS; secondary end points included PFS, ORR, and duration of response^16^. Tumor response was assessed with Response Evaluation Criteria In Solid Tumors (RECIST) version 1.0^20^ and all secondary end points were carried out by independent masked review of tumor assessments. Additionally, sensitivity analyses were completed based on investigator review.

In Study 301, OS and PFS were co-primary end points^18^. Secondary end points included ORR, duration of response, safety, quality of life, and population pharmacokinetic/pharmacodynamic relationships. Tumor response was assessed with RECIST version 1.0^20^ by independent radiology review (primary analysis) and investigator review (secondary analysis).

Findings for the primary and secondary end points of both studies have been published^16,18^.

### Post Hoc subgroup analyses

Subgroup analyses of data pooled from Studies 305 and 301 were conducted to compare the efficacy of eribulin with TPC/capecitabine according to the organ sites of metastases. Patients were divided into subgroups according to metastatic sites. The following sites were observed in >5% of all patients at baseline and were included in this analysis: bone, liver, lymph node, chest wall/breast/skin, or lung. Target and nontarget lesions were included. Of note, patients with metastases in more than 1 organ site were included in the analyses for multiple subgroups.

Analyses included OS, PFS, ORR, and percentage change in the sum of diameters of target lesions from baseline to postbaseline nadir. Moreover, analyses of OS and PFS were conducted according to ER status in each subgroup.

### Statistical methods

Analyses were conducted with pooled data and included patients in the intention-to-treat population (all patients randomly assigned to treatment groups). To determine median OS and PFS (including OS and PFS by ER status), estimates of survival were adjusted by study using methodology previously outlined in a separate pooled analysis of Studies 305 and 301^19^.

For all OS and PFS analyses, HR values and 2-sided 95% CIs were computed using Cox models with treatment as a covariate and stratified by study, region, prior capecitabine use, and HER2/neu status. Because these post hoc analyses involved multiple comparisons, all *P* values should be considered nominal and should not be used to directly determine statistical significance; nominal *P* values were based on log-rank tests and stratified as noted above. For ORR, odds ratios (95% CIs) of treatments were computed using Cochran-Mantel-Haenszel test, stratified by study. Tumor responses were based on investigator review per RESIST version 1.0.

## Results

### Patients

Patient dispositions for Study 305 and Study 301 have been reported elsewhere^16,18^. Overall, 1864 patients (Study 305, n = 762; Study 301, n = 1102) were included in this pooled post hoc subgroup analysis. Of these patients, 1062 were assigned to receive eribulin (Study 305, n = 508; Study 301, n = 554), 254 to receive TPC (Study 305), and 548 to receive capecitabine (Study 301)^16,18^. The mean dose intensity of eribulin in Study 305 was 0.78 mg/m^2^/week (standard deviation, 0.166); in Study 301, the mean dose intensity of eribulin was 0.81 mg/m^2^/week (standard deviation, 0.14), and the mean dose intensity of capecitabine was 9983.86 mg/m^2^/week (standard deviation, 1814.27).

In the pooled analysis, the median duration of treatment with eribulin was 119 days (range: 21–1372 days) and the median duration of treatment with TPC/capecitabine was 93 days (range: 1–1442 days).

Overall, the most common metastatic sites were in bone (eribulin, 57.0%; TPC/capecitabine, 58.1%) and liver (eribulin, 51.1%; TPC/capecitabine, 53.6%). Additional details regarding patient demographics and baseline characteristics have been published previously^16,18^.

### Efficacy

#### Subgroup analyses of survival by metastatic site

**Figure 1:**
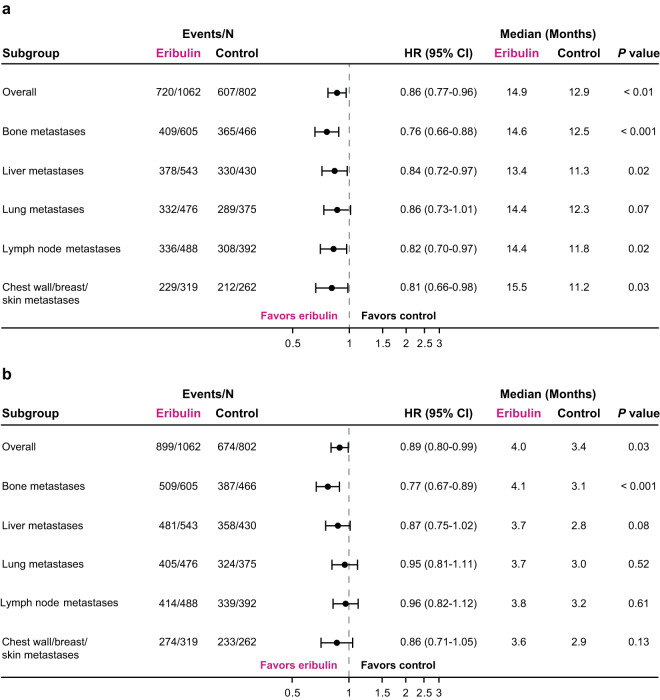
Summary of overall survival (**a**) and progression-free survival (**b**) in patients with locally advanced/MBC, by site of lesions at baseline (pooled population). (**a**) Patients randomized to eribulin had a nominally significant difference in overall survival compared with control in the overall pooled population. Patients with bone, liver, lymph node and chest wall/breast/skin metastases had a nominally significant difference in overall survival with eribulin compared with TPC/capecitabine. (**b**) Overall, patients treated with eribulin had a nominally significant difference compared with control in progression-free survival. Assessment by baseline lesion location, revealed only patients with baseline bone metastases randomized to eribulin had a nominally significant difference in progression-free survival compared with TPC/capecitabine. Control includes TPC and capecitabine; HR values and 2-sided 95% CIs were computed using Cox models with treatment as a covariate and stratified by study, region, prior capecitabine use, and HER2/neu status; nominal *P* values were based on log-rank tests and stratified as noted above. Medians were based on survival curves adjusted by study. CI, confidence interval; HR, hazard ratio; MBC, metastatic breast cancer; TPC, treatment of physician’s choice.

**Figure 2:**
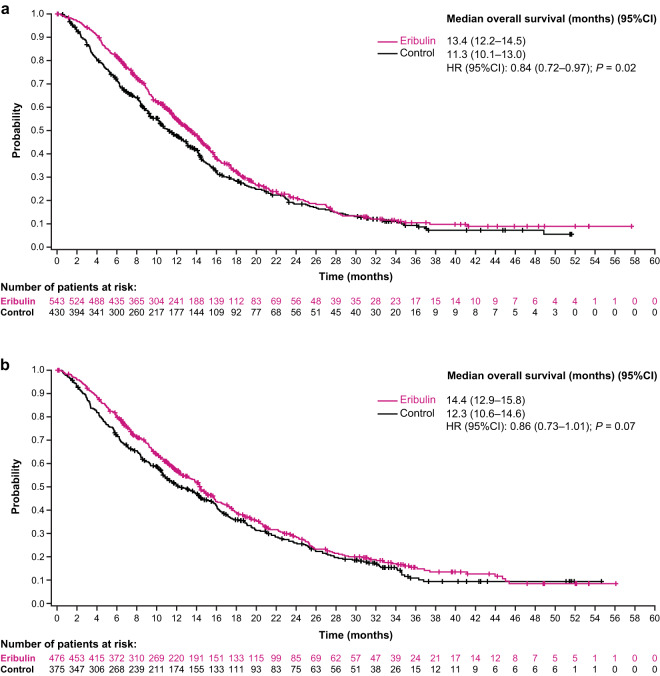
Estimated overall survival in patients with locally advanced/MBC with liver (**a**) and lung (**b**) metastases at baseline (pooled population). (**a**) Patients with baseline liver metastases randomized to eribulin had a nominally significant difference in overall survival compared with patients randomized to TPC/capecitabine. (**b**) Patients with baseline lung metastases randomized to eribulin had no significant difference in overall survival compared to control. Control includes TPC and capecitabine; HR values and 2-sided 95% CIs were computed using Cox models with treatment as a covariate and stratified by study, region, prior capecitabine use, and HER2/neu status; nominal *P* values were based on log-rank tests and stratified as noted above. Medians were based on survival curves adjusted by study. CI, confidence interval; HR, hazard ratio; MBC, metastatic breast cancer; TPC, treatment of physician’s choice.

In the overall pooled analysis, patients randomized to receive eribulin had a nominally significant difference in OS compared with patients randomized to receive TPC/capecitabine (median: 14.9 versus 12.9 months; HR, 0.86 [95% CI: 0.77, 0.96]) (Fig. 1a). In addition, OS for the eribulin group differed from the TPC/capecitabine group across all metastatic sites (Fig. 1a). Of note, patients with liver metastases who were randomized to receive eribulin had a nominally significant difference in OS compared with patients who were randomized to receive TPC/capecitabine (median:13.4 versus 11.3 months; HR, 0.84 [95% CI: 0.72, 0.97]) (Fig. 2); a nominally significant difference in OS was also observed in patients with metastases in bone (median: 14.6 versus 12.5 months; HR, 0.76 [95% CI: 0.66, 0.88]), lymph nodes (median: 14.4 versus 11.8 months; HR, 0.82 [95% CI: 0.70, 0.97]), and chest wall/breast/skin (median: 15.5 versus 11.2 months; HR, 0.81 [95% CI: 0.66, 0.98]) (Fig. 1a).

**Figure 3:**
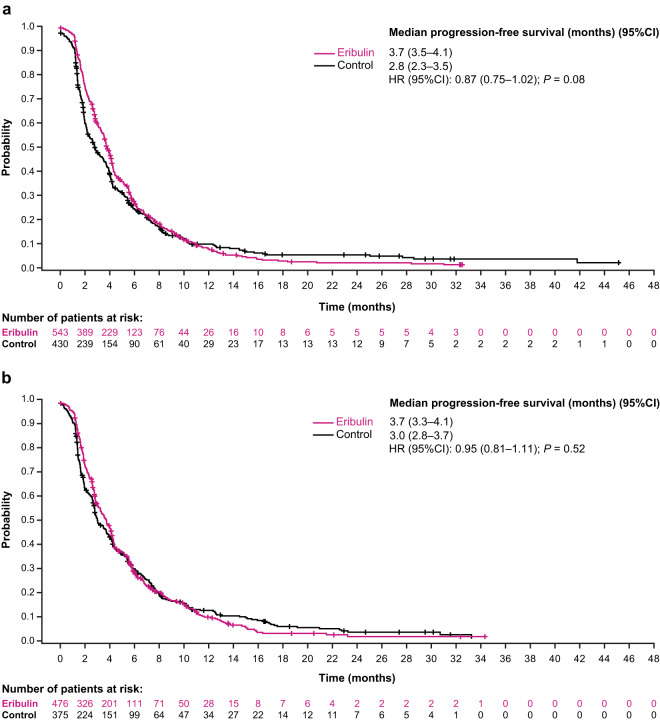
Estimated progression-free survival in patients with locally advanced/MBC with liver (**a**) and lung (**b**) metastases at baseline (pooled population). (**a**) Progression-free survival was similar in patients with baseline liver metastases regardless of treatment. (**b**) Similar results were seen in patients with baseline lung metastases. Control includes TPC and capecitabine; HR values and 2-sided 95% CIs were computed using Cox models with treatment as a covariate and stratified by study, region, prior capecitabine use, and HER2/neu status; nominal *P* values were based on log-rank tests and stratified as noted above. Medians were based on survival curves adjusted by study. CI, confidence interval; HR, hazard ratio; MBC, metastatic breast cancer; TPC, treatment of physician’s choice.

 The median overall PFS was 4.0 months for patients randomized to eribulin versus 3.4 months for patients randomized to TPC/capecitabine; the HR for progression-free survival was 0.89 (95% CI: 0.80, 0.99) (Fig. 1b). In analyses of specific subgroups, only patients with bone metastases randomized to receive eribulin had a nominally significant difference in PFS compared with patients randomized to receive TPC/capecitabine (median: 4.1 versus 3.1 months; HR, 0.77 [95% CI: 0.67, 0.89]) (Fig. 1b). PFS results were similar in the eribulin group and TPC/capecitabine group for patients with other sites of metastases, including patients with metastases in the liver (median: 3.7 versus 2.8 months; HR, 0.87 [95% CI: 0.75, 1.02]) (Figs. 1b and 3a), lung (median: 3.7 versus 3.0 months; HR, 0.95 [95% CI: 0.81, 1.11]) (Figs. 1b and 3b), lymph nodes (median: 3.8 versus 3.2 months; HR, 0.96 [95% CI: 0.82, 1.12] (Fig. 1b), and chest wall/breast/skin (median: 3.6 versus 2.9; HR, 0.86 [95% CI: 0.71, 1.05]) (Fig. 1b).

The results of these pooled analyses are generally consistent with the results of the analyses of OS and PFS by metastatic organ site at baseline in the individual Studies 305 and 301 (Online Resource Supplemental Figs. 1 and 2).

#### Subgroup analyses of survival by metastatic site and ER status

**Figure 4:**
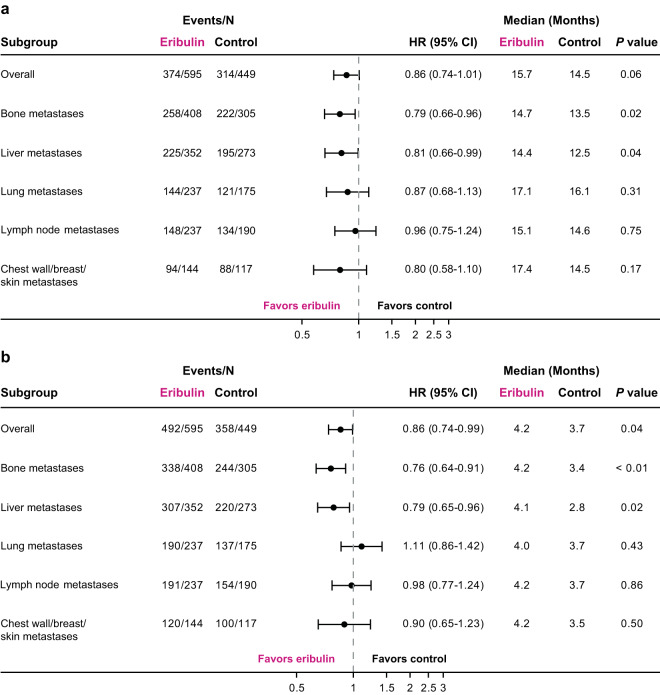
Overall survival (**a**) and progression-free survival (**b**) in estrogen-receptor–positive patients with locally advanced/MBC, by site of lesions at baseline (pooled population). (**a**) Overall survival was nominally significantly different in estrogen-receptor-positive patients with bone or liver metastases at baseline randomized to eribulin compared with TPC/capecitabine. (**b**) In the overall population, progression-free survival was nominally significantly different in estrogen-receptor-positive patients randomized to eribulin compared with control. Patients with bone or liver metastases at baseline randomized to eribulin had a nominally significant difference in progression-free survival compared with TPC/capecitabine. Control includes TPC and capecitabine. HR values and 2-sided 95% CIs were computed using Cox models with treatment as a covariate and stratified by study, region, prior capecitabine use, and HER2/neu status; nominal *P* values were based on log-rank tests and stratified as noted above. Medians were based on survival curves adjusted by study. CI, confidence interval; HR, hazard ratio; MBC, metastatic breast cancer; TPC, treatment of physician’s choice.

In patients with ER-positive disease, nominally significant differences in OS were observed with the eribulin group compared with the TPC/capecitabine group in patients with metastases to the bone (median: 14.7 versus 13.5 months, respectively; HR, 0.79 [95% CI: 0.66, 0.96]) or liver (median: 14.4 versus 12.5 months, respectively; HR, 0.81 [95% CI: 0.66, 0.99]) (Fig. 4a); nominally significant differences in PFS were also seen with metastases to the bone (median: 4.2 versus 3.4 months, respectively; HR, 0.76 [95% CI: 0.64, 0.91]) or liver (median: 4.1 versus 2.8 months, respectively; HR, 0.79 [95% CI: 0.65, 0.96]) (Fig. 4b).

**Figure 5:**
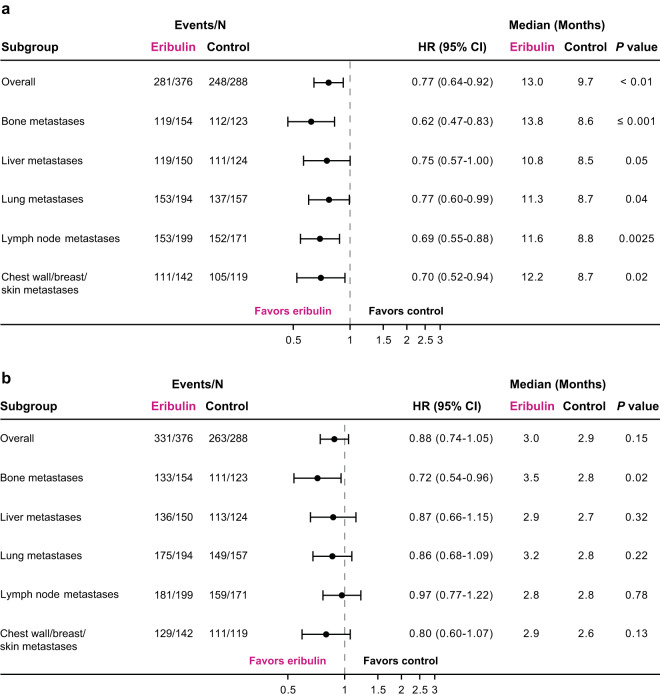
Overall survival (**a**) and progression-free survival (**b**) in estrogen-receptor–negative patients with locally advanced/MBC, by site of lesions at baseline (pooled population). (**a**) Overall survival was nominally significantly different in estrogen-receptor-negative patients in the overall population, and in patients with bone, lung, lymph node or chest wall/breast/skin metastases at baseline randomized to eribulin compared with TPC/capecitabine. (**b**) Progression-free survival was nominally significantly different only in estrogen-receptor-negative patients with bone metastases at baseline randomized to eribulin compared with TPC/capecitabine. Control includes TPC and capecitabine; HR values and 2-sided 95% CIs were computed using Cox models with treatment as a covariate and stratified by study, region, prior capecitabine use, and HER2/neu status; nominal *P* values were based on log-rank tests and stratified as noted above. Medians were based on survival curves adjusted by study. CI, confidence interval; HR, hazard ratio; MBC, metastatic breast cancer; TPC, treatment of physician’s choice.

In patients with ER-negative disease, the eribulin group had nominally significant differences in OS compared with the TPC/capecitabine group in patients with metastases to the bone (median: 13.8 versus 8.6 months, respectively; HR, 0.62 [95% CI: 0.47, 0.83]), lung (median: 11.3 versus 8.7 months, respectively; HR, 0.77 [95% CI: 0.60, 0.99]), lymph nodes (median: 11.6 versus 8.8 months, respectively; HR, 0.69 [95% CI: 0.55, 0.88]), or chest wall/breast/skin (median: 12.2 versus 8.7 months, respectively; HR, 0.70 [95% CI: 0.52, 0.94]); a numerical difference in OS was observed in patients with liver metastases (median: 10.8 versus 8.5 months; HR, 0.75 [95% CI: 0.57, 1.00]) (Fig. 5a). Only patients with ER-negative disease and bone metastases had a nominally significant difference in PFS (median: 3.5 versus 2.8 months; HR, 0.72 [95% CI: 0.54, 0.96]) with randomization to eribulin versus TPC/capecitabine (Fig. 5b).

#### Tumor response according to sites of metastases at baseline

**Figure 6:**
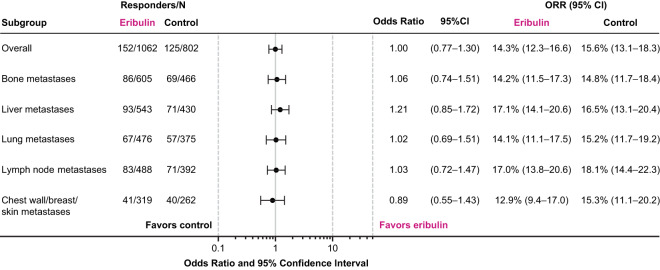
Summary of tumor response as assessed by investigators per RECIST version 1.0, by site of lesions at baseline (pooled population). The overall response rates were similar across the pooled population regardless of location of metastases at baseline. Control includes TPC and capecitabine; odds ratio (95% CI) was calculated using Cochran-Mantel-Haenszel test, stratified by study. CI, confidence interval; RECIST, Response Evaluation Criteria In Solid Tumors; TPC, treatment of physician’s choice.

ORR was similar for patients randomized to eribulin (14.3% [95% CI: 12.3, 16.6]) and TPC/capecitabine (15.6% [95% CI: 13.1, 18.3] in the overall pooled population (Fig. 6). Individual analyses of ORR by site of metastases at baseline in Studies 305 and 301 are included in Online Resource Supplemental Figs. 3 and 4. A nominally significant difference in ORR between the treatment groups was observed for the overall population, bone metastases, and liver metastases in Study 305 (Online Resource Supplemental Fig. 3). For Study 301, none of the observed differences in ORR were nominally significant (Online Resource Supplemental Fig. 4).

#### Analyses of percentage change in sum of diameters of target lesions from baseline to postbaseline nadir by metastatic site

**Figure 7:**
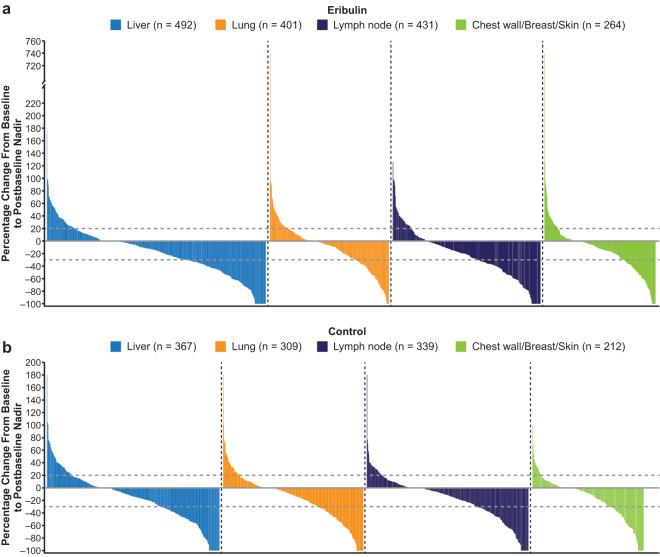
Percentage change in sum of diameters of target lesions from baseline to postbaseline nadir, by metastatic site at baseline in patients randomized to receive eribulin (**a**) or control (**b**) (pooled population). Patients with liver, lung, lymph node or chest wall/breast/skin metastases showed decreased tumor sizes when treated with (**a**) eribulin or (**b**) TPC/capecitabine. n is the number of patients with both baseline and postbaseline measurements of target lesions.

Among patients with both baseline and postbaseline measurements of target lesions, 63% (544/857) randomized to receive eribulin and 63% (403/638) randomized to receive TPC/capecitabine experienced a reduction in tumor size in postbaseline nadir imaging studies (ie, percentage change in the sum of the diameters of the target lesions from baseline to postbaseline nadir imaging studies showed a reduction in tumor size). Of patients with liver metastases randomized to receive eribulin or TPC/capecitabine, 66% (n = 325/492) and 63% (n = 231/367) respectively, had reductions in tumor size on postbaseline nadir imaging studies (Fig. 7). Similar results for eribulin versus TPC/capecitabine were seen for patients with lung metastases (63% [n = 252/401] versus 61% [n = 190/309]), lymph node metastases (68% [n = 294/431] versus 65% [220/339]), and chest wall/breast/skin metastases (66% [n = 173/264] versus 62% [n = 131/212]) (Fig. 7).

## Discussion

The efficacy of eribulin in locally advanced/MBC has been previously established in Studies 305 and 301, which demonstrated a statistically significant improvement in OS compared with TPC (Study 305: *P* = 0.041)^16^, and similar OS compared with capecitabine (Study 301: *P* = 0.056)^18^. The results from this post hoc exploratory pooled analysis are largely consistent with these findings. Our current analyses provide insights into the efficacy of eribulin with regard to the organ sites of metastases at baseline. Such insights are of potential relevance because a patient’s prognosis is dependent on the location of metastases at baseline. Specifically, patients with visceral disease (eg, metastases in the lung, liver, brain) are known to have reduced survival duration^4^, and among patients with visceral disease, metastases to the liver (median OS, 13 months) or brain (median OS, 7 months) generally portend the worst prognosis^21^.

The pooled analysis presented here suggests that eribulin may be an effective treatment option in patients with locally advanced/MBC, irrespective of their organ sites of metastatic disease at baseline (ie, bone, liver, lung, lymph nodes, and chest wall/breast/skin). In this analysis, nominal differences in OS (*P* = 0.02) were observed in favor of eribulin versus TPC/capecitabine in patients with liver metastases at baseline. ORRs were similar with eribulin (17.1%) and TPC/capecitabine (16.5%) in this population. Given the typically poor prognosis of patients with chemotherapy-pretreated bone, liver, lung, lymph nodes, and chest wall/breast/skin metastases, the efficacy of eribulin in these patients is noteworthy. Moreover, the ability of eribulin to reduce target lesion tumor size may be clinically significant in reducing metastases-associated symptoms and organ dysfunction.

The consistent efficacy of eribulin across metastatic sites is likely the result of its mechanism of action (ie, its unique interaction with microtubules)^8–11^. Eribulin’s efficacy against difficult-to-treat liver metastases, in particular, may be the result of a multifaceted interplay between eribulin’s mechanism of action and the complex microenvironment of the liver. Specifically, the potential for eribulin to reverse EMT and inhibit cancer cell migration/invasion may provide a mechanistic advantage for eribulin in the treatment of liver metastases, given that liver sinusoidal endothelial cells have been shown to induce EMT in colorectal cancer cells^12–14,22^.

Of interest, the absolute prolonged median OS time difference with eribulin compared with control was 2.0 months (eribulin, 14.9 months; control, 12.9 months), whereas the absolute prolonged time of median PFS difference with eribulin compared to control was 0.6 months (eribulin, 4.0 months; control, 3.4 months). Previous studies with eribulin have suggested that the discordance between OS and PFS improvement may be partly due to eribulin’s ability to remodel the tumor microenvironment and vasculature; such remodeling may improve the antitumor activity of eribulin, as well as positively impact the efficacy of subsequent anticancer therapies, resulting in improved OS^13,23^. Additionally, a previous study by Twelves *et al*.^24^ indicated that the development of new metastasis is a risk factor for shorter OS. As both growth of existing metastasis and development of new metastasis are considered “progression” in terms of PFS, whereas the more aggressive form of progression (ie, development of new metastasis) is considered a risk factor for shorter OS, the difference between these forms of progressive disease may not be captured by the PFS end point but instead may be reflected in the OS end point. Moreover, in support of this theory, Twelves *et al*. showed that new-metastasis-free survival was longer in the eribulin arm than in the comparator arm in EMBRACE, which could account for the improved OS seen with eribulin treatment^24^. This may provide a rationale for the more prolonged OS, but not PFS, observed in eribulin-treated patients in the current analysis.

This analysis is limited by the relatively small number of patients in each subgroup, its post hoc nature, and the multiple testing comparisons; as such, *P* values are considered nominal. Despite these limitations, the results of this post hoc analysis are consistent with previous findings of eribulin’s efficacy in locally advanced/MBC^16,18^, and suggest that eribulin can be an effective treatment option in patients with bone, liver, lung, lymph node, and chest wall/breast/skin metastases.

## Supplementary information

Supplementary information.

## Data Availability

The datasets generated during and/or analyzed during the current study are not publicly available, but are available from the corresponding author on reasonable request. However, information that could be used to identify patients are not available, nor was any such information available to any of the authors.
