# Supplementary material for: Efficacy of eribulin for metastatic breast cancer based on localization of specific secondary metastases: a post hoc analysis
Source: Sci Rep. 2020 Jul 8;10:11203. doi: 10.1038/s41598-020-66980-0 (PMC7343788; doi:10.1038/s41598-020-66980-0)
Supplement: Supplementary file 1 — Supplementary information. [file 41598_2020_66980_MOESM1_ESM.docx]

**Supplementary section**

**Efficacy of eribulin for metastatic breast cancer based on localization of specific secondary metastases: a post hoc analysis**

**Authors:** Joyce O’Shaughnessy,^1^ Javier Cortes,^2^ Chris Twelves,^3^ Lori J. Goldstein,^4^ Karenza Alexis,^5^ Ran Xie,^5^ Carlos Barrios,^6^ Takayuki Ueno^7^

**SUPPLEMENTARY SECTION**

**Online Resource Supplemental Fig. 1** Summary of overall survival in patients with MBC (a) and progression-free survival (b), by site of lesions at baseline (Study 305)


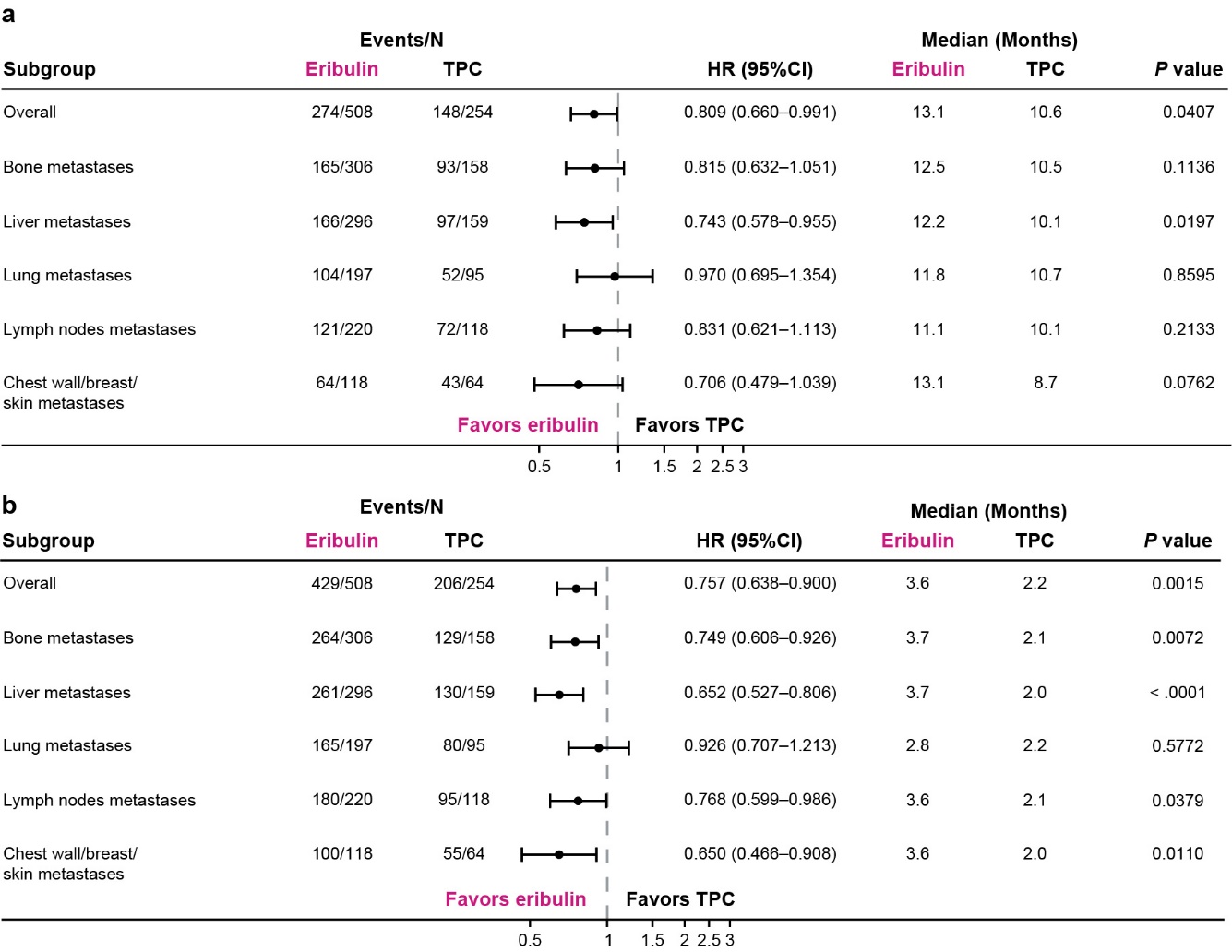


In Study 305, patients randomized to eribulin had a nominally significant difference in overall survival compared with patients randomized to TPC in the overall population (a). Patients with liver metastases also had nominally significant differences in overall survival when randomized to eribulin compared with TPC. (b) Overall, patients randomized to eribulin had a nominally significant difference in progression-free survival compared with TPC. Patients with bone, liver, lymph node and chest wall/breast/skin metastases also had better progression-free survival with eribulin compared with control.

For the overall population, HR value and 2-sided 95% CIs were computed using Cox models with treatment as a covariate with HER2/neu status, prior capecitabine treatment, and geographical region as strata. Nominal *P* value was based on log-rank test. No strata were used for analyses in subgroups.

CI, confidence interval; HR, hazard ratio; TPC, treatment of physician’s choice.

**Online Resource Supplemental Fig. 2** Summary of overall survival in patients with MBC (a) and progression-free survival (b), by site of lesions at baseline (Study 301)


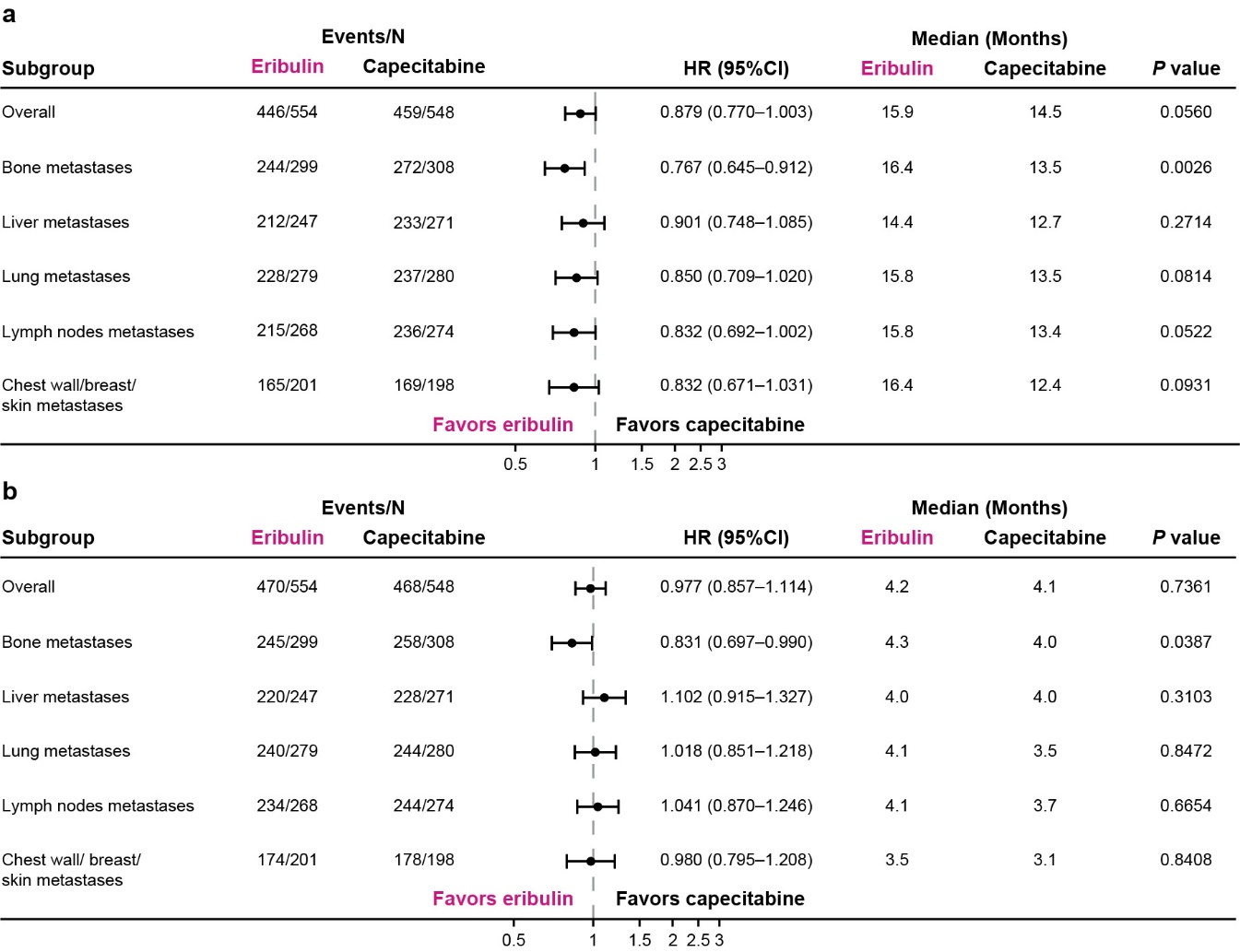


In Study 301, (a) patients with bone metastases at baseline and randomized to eribulin had a nominally significant difference in overall survival compared with capecitabine. (b) Patients with bone metastases randomized to eribulin also had a nominally significant dfference in progression-free survival compared with capecitabine.

For the overall population, HR values and 2-sided 95% CIs were computed using Cox models with treatment as a covariate with HER2/neu status and geographical region as strata. Nominal *P* values was based on log-rank test and stratified as noted above. No strata were used for analyses in subgroups.

CI, confidence interval; HR, hazard ratio; MBC, metastatic breast cancer.

**Online Resource Supplemental Fig. 3** Summary of tumor response as assessed by investigators per RECIST version 1.0, by site of lesions at baseline (Study 305)


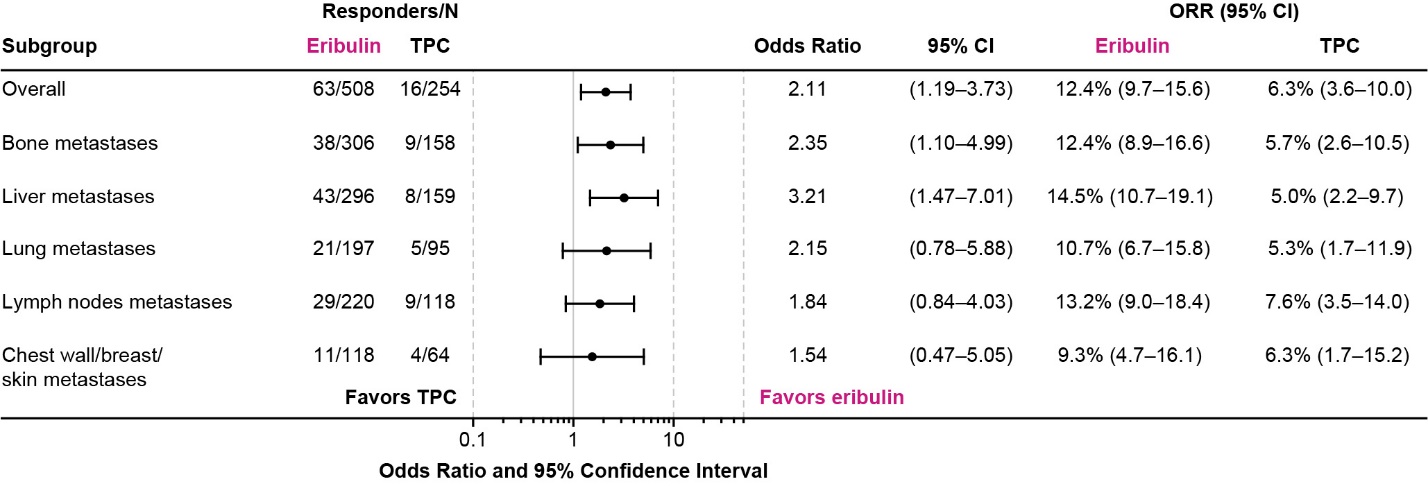


In Study 305, a nominally significant difference in the overall response rate was observed for the overall population, patients with bone metastases, and patients with liver metastases for the eribulin group compared with the TPC group.

For the overall population, the odds ratio and its 95% CIs were computed using the Cochran-Mantel-Haenszel test with HER2/neu status, prior capecitabine treatment, and geographical region as strata. No strata were used for analyses in subgroups.

CI, confidence interval; HR, hazard ratio; ORR, objective response rate; RECIST, Response Evaluation Criteria In Solid Tumors; TPC, treatment of physician’s choice.

**Online Resource Supplemental Fig. 4** Summary of tumor response as assessed by investigators per RECIST version 1.0, by site of lesions at baseline (Study 301)


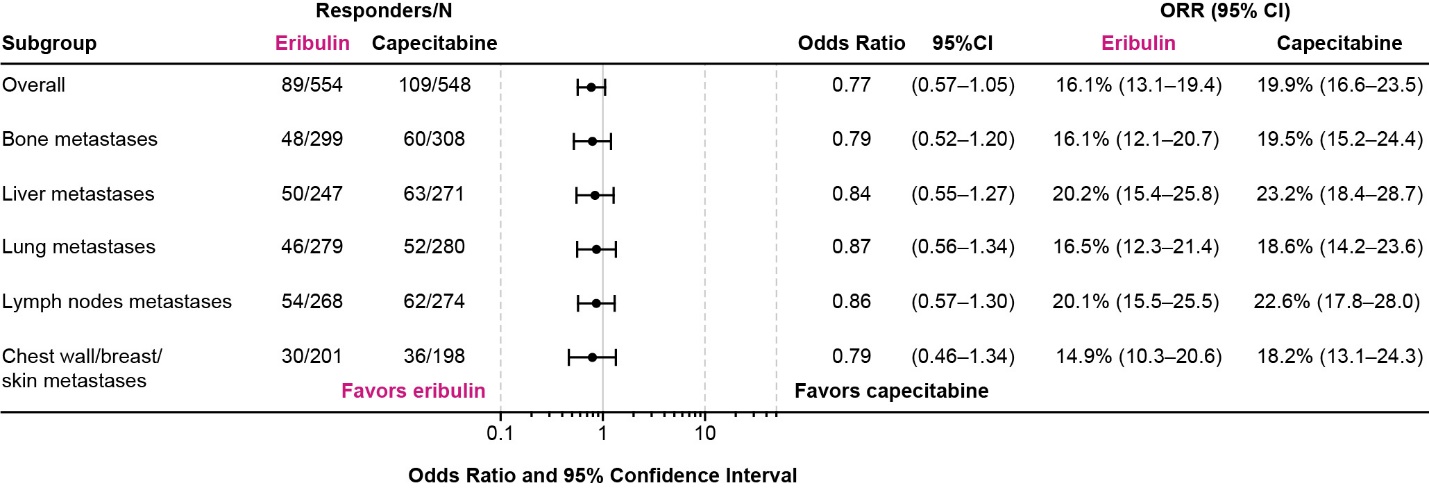


In Study 301, none of the observed differences in the overall response rate were nominally significant.

For the overall population, the odds ratio and its 95% CIs were calculated using the Cochran-Mantel-Haenszel test with HER2/neu status and geographical region as strata. No strata were used for analyses in subgroups.

CI, confidence interval; HR, hazard ratio; ORR, objective response rate; RECIST, Response Evaluation Criteria In Solid Tumors.
